# Blood Aspergillus PCR: The Good, the Bad, and the Ugly

**DOI:** 10.3390/jof6010018

**Published:** 2020-01-27

**Authors:** Matthias Egger, Jeffrey D. Jenks, Martin Hoenigl, Juergen Prattes

**Affiliations:** 1 Section of Infectious Diseases and Tropical Medicine, Medical University of Graz, 8036 Graz, Austria; matthias.egger@stud.medunigraz.at (M.E.); martin.hoenigl@medunigraz.at (M.H.); 2 Department of Medicine, University of California San Diego, San Diego, CA 92093, USA; jjenks@health.ucsd.edu; 3 Clinical and Translational Fungal Research Group, University of California San Diego, San Diego, CA 92093, USA

**Keywords:** *Aspergillus*, invasive aspergillosis, diagnosis, PCR, blood, antifungal prophylaxis

## Abstract

Invasive *Aspergillosis* (IA) is one of the most common invasive fungal diseases and is accompanied by high morbidity and mortality. In order to maximize patient outcomes and survival, early and rapid diagnosis has been shown to be pivotal. Hence, diagnostic tools aiding and improving the diagnostic process are ambitiously searched for. In this context, polymerase chain reaction (PCR) may represent a potential candidate. Its additional value and benefits in diagnosis have been demonstrated and are scientifically established. Nevertheless, standardized and widespread usage is sparse because several factors influence diagnostic quality and need to be considered in order to optimize diagnostic performance and outcome. In the following review, the current role of PCR in the diagnosis of IA is explored, with special focus on the strengths and limitations of PCR in different settings.

## 1. Blood Aspergillus PCR

### 1.1. Introduction

Polymerase chain reaction (PCR)-based diagnostic methods revolutionized medicine. However, the exact role of PCR in diagnosing invasive fungal infections (IFIs) is still under debate, given the lack of standardization and large variation of diagnostic performance across studies and settings. Several PCR-based methods are currently used in routine clinical practice to diagnose or rule out suspected IFI. Performance of blood PCR methods are highly dependent on patient cohort characteristics including accompanying pretest probability and antifungal prophylaxis, and also PCR characteristics, such as number of cycles and limit of detection. A reliable diagnostic assay is specifically needed for IA, as IA remains associated with high morbidity and mortality [1,2]. IA remains difficult to diagnose using culture-based methods given the low sensitivity of *Aspergillus* recovery from blood and bronchoalveolar lavage fluid (BALF) [3]. Consequently, antigen testing—in particular galactomannan (GM) testing—has emerged as the gold-standard for diagnosing IA. As performance of GM testing is still less than perfect and turnaround time varies [4,5], the search for improved biomarker tests continues, with the focus on point-of-care lateral flow device tests [6,7,8], immunologic markers, markers of iron acquisition [9,10], and PCR testing [1]. Even though IA remains the predominant mold infection in immunocompromised hosts, its prevalence remains low at 0.2% of hospital admissions among patients considered to be at risk for IFIs [11]. The low prevalence of IA, together with often non-specific clinical or radiologic signs of IA [12], pose significant challenges to diagnostic strategies.

While blood PCR has been used as an aid in the diagnosis of IA for almost three decades, broad implementation has been limited given the lack of standardization of PCR assays [13] and variable performances in different settings [14,15]. Recent guidelines for the diagnosis and management of *Aspergillus* disease moderately recommended PCR on blood samples for the diagnosis of IA, with a quality of evidence (QoE) Level II [16], and very recently PCR has also been included as mycological criterion in consensus definitions of IA [17]. Despite these advances, blood PCR is still not used widely in clinical routine. In this critical review of available literature we will try to clarify the role of blood PCR for diagnosing IA, focusing on its strengths (“the good”), limitations (“the bad”), and outlining clinical settings and patient cohorts where performance of blood PCR has been largely disappointing (“the ugly”).

### 1.2. The Good

One of the major strengths of blood *Aspergillus* PCR is its high sensitivity and negative predictive value (NPV) in severely immunocompromised patients with underlying hematological malignancies in settings where antifungal prophylaxis is not used. A 2019 published systematic review highlighted a mean sensitivity and specificity of 79.2% and 79.6% for a single positive test result and 59.6% and 95.1% for two consecutive positive results when performing PCR on blood samples (serum or whole blood). The majority of investigated cohorts were hematological malignancy patients at highest risk for IA (i.e., neutropenic cancer patients, hematopoietic stem cell transplant (HSCT) recipients) [18]. In this setting with high prevalence of 16.3%, the NPV was 95.1% for one negative test result and 92.4% for two negative test results [18]. Higher NPVs are therefore expected in a lower prevalence setting, indicating that a single PCR-negative test result may be adequate to exclude a diagnosis of IA.

Another benefit of blood PCR is its higher potential to diagnose IA earlier compared to GM. Several studies have shown that *Aspergillus* DNA in the bloodstream precedes the release of GM into the bloodstream and thus PCR positivity is indicative of *Aspergillus* exposure prior to other biomarkers becoming detectable or clinical signs or symptoms of the disease being noticeable [19,20]. As a consequence, positive test results, particularly when PCR is combined with other molecular diagnostic tests such as GM (increase of specificity), may be used to initiate further diagnostic work-up. The latter includes bronchoscopy with biopsy or BALF, and eventually initiation of antifungal therapy [21,22]. Although *Aspergillus* DNA correlates quantitatively to serum GM levels [23], it is important to remember that at different stages of invasive disease different antigens may be detectable in patient blood [24]. This highlights the importance of combining different biomarkers in the diagnostic process and to repeat available diagnostic tests if they turn out negative, but clinical suspicion of IA is still high. In high-risk settings that don’t use mold-active antifungal prophylaxis, screening strategies with serum GM and blood PCR could therefore represent an alternative to empiric antifungal treatment strategies (i.e., based on neutropenic fever not responsive to antibacterial therapy), reducing the use and cost of antifungal agents. Additionally, potentially unnecessary drug exposure and toxicity, associated with severe side effects, could be avoided [25,26]. In contrast, screening for IA is not recommended in settings that use mold-active prophylaxis, which has been shown to be highly effective in those at the highest risk for developing IA, decreasing IA prevalence to 3% and below [27,28,29]. This may be due to the low prevalence of IA in those settings, which reduces the positive predictive value (PPV) of a positive PCR test result, as well as the fact that antifungals may inhibit PCR and therefore limit diagnostic utility, as discussed in more detail later in this review [30,31]. The same limitations also apply for serum GM testing [32]. Thus, general screening for IA in patients on mold-active prophylaxis is currently not recommended, where both PCR and GM should instead be used for targeted testing in the case of clinical or radiological suspicion of breakthrough IA [5,27].

*Aspergillus fumigatus* (*A. fumigatus*) is the most common cause for IA [33,34] and most prior studies have focused on the evaluation of PCR assays for this species. Epidemiology of IA globally varies and species other than *A. fumigatus* are increasing in prevalence [35]. Additionally, non-*Aspergillus* molds occur more frequently due to the increasing use of antifungal prophylaxis and more intensive immunosuppressive therapies [36]. As a result, it is important that *Aspergillus* PCR assays show a high analytical specificity and are capable of differentiating between several *Aspergillus* species without cross-reacting to other molds, because non-*Aspergillus* molds may require different anti-fungal treatment and some *Aspergillus* species can be intrinsically resistant to polyenes or azoles [37]. To date, *Aspergillus* PCR is the only non-culture-based assay that is able to differentiate *Aspergillus* spp. down to the species level. However, due to inferior detection of *Aspergillus* species other than *A. fumigatus*, current recommendations prefer the use of genus-specific PCR assays rather than species-specific ones [38].

A significant strength of some blood *Aspergillus* PCR assays is their ability to detect major single nucleotide polymorphisms, implying environmentally acquired resistance [39]. Increasing *Aspergillus* resistance to antifungal drugs is an emerging problem in clinical practice in some parts of the world [40,41,42]. As stated above, resistance can be intrinsic, but the problem of increasing resistance is mostly driven by environmentally acquired resistance due to exposure to azole compounds, either in clinical settings or—more often—in agriculture where azoles are used commonly as fungicides [43]. Acquired azole resistance is primarily found in *A. fumigatus*. Most azole resistance patterns are caused by point mutations in the *cyp51A* gene. Two commercially available PCR kits are able to detect the most relevant point mutations in this gene and therefore azole resistance, namely the AsperGenius^®^ and MycoGENIE^®^, with similar diagnostic performance [44]. However, current data is insufficient and further evaluation of assays targeting resistance genes is required.

Whether serum or whole blood samples should be used to perform PCR remains a matter of debate. While sensitivities reported in studies using serum samples were comparable to those using whole blood samples [45,46,47], there are studies indicating a trend towards higher sensitivity of whole blood/plasma samples compared to serum samples [45,48]. A possible explanation for the higher sensitivity in plasma/whole blood samples is the higher amount of cell-free DNA in plasma samples compared to serum samples. This may be the consequence of clotting in serum samples as DNA concentrations in clots were found to be higher than those in serum, leading to further decrease and a lower abundance of circulating *Aspergillus* DNA in serum during invasive disease [49,50]. Still, PCR testing from serum has the advantage that other biomarkers for IA like galactomannan can be measured in the same sample and thus reducing the amount of blood drawn.

In summary, evidence supports that for the diagnosis of IA in neutropenic patients, *Aspergillus* PCR in blood is as good as serum GM and superior to 1,3-beta-d-glucan, which cross reacts with *Candida* spp. and may be elevated in various conditions associated with fungal translocation from the gut [51]. Another benefit of PCR is the potential detection of acquired azole resistance. Consequently, PCR was recently included in the new European Organization for the Research and Treatment of Cancer (EORTC)/Mycoses Study Group (MSG) consensus definitions of IA [17].

### 1.3. The Bad

While *Aspergillus* PCR is now well established for use in neutropenic patients with underlying hematological malignancies in settings that do not use mold-active prophylaxis, its usefulness in other settings remains unclear. Importantly, the pathogenesis of IA differs between neutropenic and non-neutropenic patients [52,53], impacting clinical presentation, radiological findings, and diagnostic test results in the mycology laboratory [54]. Neutropenic patients usually develop angio-invasive disease associated with increased serum GM levels, but sensitivities of serum GM decrease to 30% and less in non-neutropenic patients who primarily develop tissue invasive disease with only limited angio-invasion [52,53,55,56,57]. In contrast, BALF GM testing remains reliable independent of neutrophil count, as the lungs are usually the primary site of infections caused by *Aspergillus* [1,58,59,60]. Similarly, PCR testing of blood specimens seems to have limited diagnostic ability in non-neutropenic patients, with sensitivities as low as 11% in intensive care unit (ICU) patients [61], while sensitivity and overall diagnostic performance of BALF PCR testing has been shown to be significantly better, reaching sensitivities of 44%–100% [61,62,63,64]. Even in patients with hematological malignancies but without neutropenia, *Aspergillus* PCR from blood samples proved to be insufficiently sensitive to be diagnostically useful after day 100 post stem cell transplantation [65]. As patients after allogeneic stem cell transplantation may develop graft-versus-host disease and therefore require long-term immunosuppressive treatment putting these patients on risk for IA for a long period of time, the utility of blood PCR for diagnosis of IA after engraftment is questionable. The limited utility of PCR from blood specimens in non-neutropenic patients is a very relevant limitation given that the prevalence of IA continues to increase in non-neutropenic patients with other severe underlying diseases. This includes patients in the ICU where prevalence rates vary between 0.33%–19% [66,67,68,69], solid organ transplant recipients [70], patients receiving systemic glucocorticoids [71], patients with underlying respiratory conditions [66,72], patients with solid cancers [66,73], and other patient groups [66,74].

Positive *Aspergillus* PCR in BALF samples should also be interpreted with caution and cannot be used as a sole marker for the presence of IA. This is due to the fact that different cohorts of patients with structural or functional lung disease are likely to be colonized with *Aspergillus* in the lower respiratory tract but to not necessarily develop invasive disease as long as no additional immunosuppressive disease or treatment is present [75,76,77,78,79]. In such cases, a positive BALF PCR result may represent either colonization of the patient or actual invasive disease. To discriminate these two entities in a patient with a positive lower respiratory tract, *Aspergillus* PCR, clinical presentation, imaging studies, and other biomarkers are helpful. Contamination of the obtained specimen during sampling or during processing in the laboratory may also cause a false positive PCR result. 

Importantly, standardization of blood PCR remains a tricky challenge and complicates comparison and generalization of study results. While a variety of commercially available PCR assays are currently in use, only a few have been investigated in large cohort studies (e.g., AsperGenius^®^, MycAssay *Aspergillus*^®^, MycoGENIE^®^) [44]. The mentioned assays have shown comparable results in different studies. For BALF samples in hematological patients, sensitivities and specificities were reported reaching from 80% to 92.9% and 80% to 97.1% [80,81,82]. In particular, sensitivity has been shown to be significantly lower in serum samples compared to respiratory samples [83]. 

The absence of general recommendation on which PCR tests are preferable and the existence of a vast number of in-house assays, most of which are lacking external validation, is restraining the use of PCR in many countries and impairing standardization. Not only the nature of samples studied (whole blood, serum, plasma) and volume of sample tested, but also specimen processing and DNA extraction methods, gene targets, amplification platforms, detection methodologies, and definitions of PCR positivity differ widely between these PCR assays. While some assays rely on a high number of amplification circles to lower the limit of detection to below one colony forming unit (CFU)/mL and thereby increase sensitivity, this comes at the cost of specificity, potentially driven by low-level fungal translocation which may occur in a variety of diseases [51,84,85,86,87,88]. Standardization in terms of defining a positive PCR is therefore needed. Technical methodology such as time to reporting, access to testing centers, and frequency of testing also needs to be optimized in order to acquire reliable results within a reasonable turnaround time [89]. Facing these issues and tackling specifically extraction procedures of nucleic acid which are crucial [90], the European *Aspergillus* PCR initiative (EAPCRI) was founded with the aim of standardizing *Aspergillus* PCR methodology [91]. While this was a step in the right direction, widespread acceptance and standardization is still lacking. 

### 1.4. The Ugly

The good diagnostic performance and ability to detect azole resistance makes *Aspergillus* PCR from blood specimens an attractive option for screening and diagnosis in neutropenic patients, while lack of standardization and bad performance in non-neutropenic patients are the most important drawbacks. There is one additional development that turns out to be the ugliest enemy of broad implementation of *Aspergillus* PCR from blood specimens, namely the advent of mold-active antifungal prophylaxis. More than a decade ago, mold-active prophylaxis was shown to significantly decrease the prevalence of IFI and increase overall survival in patients with acute leukemia with prolonged neutropenia undergoing induction chemotherapy [29,32,92]. Reflecting this evidence, all major international guidelines now strongly recommend mold-active prophylaxis for this setting [16,28,93], and the prophylaxis strategy has now been adapted in most centers that treat patients with hematological malignancies. This development turned out ugly for *Aspergillus* PCR from blood specimens because of its poor performance in patients on systemic mold-active prophylaxes (Table 1). Only a few studies explicitly provide the exact number of patients receiving antifungal treatment. Knowledge of the number of patients receiving antifungal prophylaxis is important in order to reliably compare diagnostic performance of *Aspergillus* PCR (see Table 1). A clear difference in settings applying antifungal treatment versus settings with no antifungal treatment is noticeable and given the low sensitivity and specificity of *Aspergillus* PCR in this population, its use is probably not supported in these circumstances.

In fact, *Aspergillus* PCR from blood specimens is unable to be used to diagnose breakthrough IA, with sensitivities as low as 8% and 0% for probable and proven IA in single blood samples obtained on the day of diagnostic bronchoscopy [62]. One may argue that due to the low fungal burden in early breakthrough infections, the threshold of PCR assays needs to be lowered in order to make detection possible. However, in another recent study in a setting where 100% of patients were receiving mold-active prophylaxis/treatment lowering the cutoff produced a reasonable sensitivity, but this came at the cost of unacceptable low specificities of 27.4% (95% CI 17.6%–39.1%) for one positive and 52.1% (95% CI 40.0%–63.9%) for two positive *Aspergillus* PCR test results (15). The reasons for these strikingly low specificities observed at those very low cut-offs are not fully understood, but several factors may play a part. Importantly, while *Candida* spp. are predominant, *Aspergillus* spp. also represent a relevant component of the gut mycobiome [94], detectable in 32% of studies and 24% of samples [95]. Low-level translocation of fungal components from the gut may remain undetected in blood culture but may trigger false positive PCR results after multiple amplification cycles [84,96]. In addition, antifungal agents may increase the load of fungal DNA during the destruction of fungal cells, resulting in a large load of fungal DNA and thus lowering specificity.

In contrast to blood PCR, *Aspergillus* PCR from BALF shows reasonable diagnostic performance in patients receiving mold-active prophylaxis. According to recently published studies, the sensitivity of PCR when applied to same-day BALF samples was significantly higher when compared to blood samples reaching 44%–63% in patients on mold-active prophylaxis, with close to perfect specificity [62]. The use of PCR in prophylaxis settings may be restricted to the detection of breakthrough infections and monitoring the response to therapy. Evidence for this usage is scant.

## 2. Conclusions

The diagnostic quality of *Aspergillus* PCR from blood is heavily dependent on a number of influencing variables and therefore varies between settings. When used in the right patient cohort, *Aspergillus* PCR from blood specimens may help diagnose or rule out IA. Due to its high NPV, PCR may be used as a screening tool in neutropenic patients at high risk for IA and who are not receiving mold-active prophylaxis. If there are no specific clinical signs of IA in conjunction with a single negative PCR test result, empirical antifungal therapy can possibly be safely withheld, resulting in a reduction of unnecessary use of antifungal agents and therefore a lower number of patients exposed to potential drug toxicity. Regarding the role of PCR from blood specimens for confirming IA diagnosis in neutropenic patients, recent studies have shown encouraging results when PCR is combined with other antigen-based biomarkers such as GM. Consequently, positive PCR results show very high specificity and can be used to trigger further diagnostic work-up or administration of empirical antifungal therapy when infection is highly suspected. A combination of GM and PCR is preferable due to improved diagnostic accuracy and the potential for more rapid diagnosis. Drawbacks of PCR testing include lack of standardization, although things are improving since the EAPCRI started tackling this problem. Thus, future research in terms of fungal PCRs is supposed to further address a standardization of PCR assays to ensure comparability of study results and therefore enable the evaluation of PCR performance in a significantly larger number of patients. This would also increase the number of patients with proven IA, a usually underrepresented classification of IA in most studies, as most patients are classified as having probable IA based on clinical and radiological signs of IA combined with a positive GM test. 

In contrast, diagnostic utility of *Aspergillus* PCR from blood specimens is generally limited outside the hematological malignancy setting, where BALF PCR is usually superior. The biggest hurdle to broad implementation of *Aspergillus* PCR from blood specimens, however, is its poor diagnostic performance in the presence of mold-active prophylaxis, which is now the standard of care in most centers around the world in several patient settings. These developments largely limit the application of *Aspergillus* PCR from blood specimens in those at highest risk. However, there are still areas remaining where the test can be useful, particularly in neutropenic patients at moderate risk for IA for whom antifungal prophylaxis is not (yet) recommended, including patients who develop prolonged neutropenia during induction chemotherapy for acute lymphoid leukemia, or chemotherapy for other hematologic malignancies.

## Figures and Tables

**Table 1:** Performance of *Aspergillus* PCR in blood comparing settings with no antifungal prophylaxis versus settings with antifungal prophylaxis.

Author	Patients Characteristics	Patients (Samples)	IPA Cases	Proportion of Patients on Antifungals at Time of PCR	Material	PCR Assay	Cut off/Cycles	Sensitivity % (95% CI)	Specificity % (95% CI)	NPV %/NLR	PPV%/PLR	DOR	Reference
Cesaro et al.	Hematological malignancies	62 (536)	8 *	42%	Whole blood–single ^§§^	Real time PCR	45	88	37	95	17	-	[97]
					Whole blood-multiple ^§§^			63	81	94	33		
Eigl et al.	Hematological malignancies	53 (53)	16 *	64%	Whole blood	Nested	-	0 (0–19.4)	100 (90.6–100.0)	70	NA	NA	
Boch et al.	Hematological malignancies	133 (138)	38 *	67%	Whole blood	Nested	-	8 (3–20)	87 (70–95)	1.07 ^1^	0.58 ^2^	0.54	[62]
Hummel et al.	Hematological malignancies	91 (459)	30 *	70%	Whole blood	Nested	1–5 CFU/mL	43	-	-	-	-	[98]
Heldt et al.	Hematological malignancies	106 (106)	11 *	80%	Serum	Nested	-	0 (0–27.8)	100 (93.5–100.0)	85	NA	NA	[99]
Aslan et al.	Hematological malignancies	99 (358)	18 *	90%	Serum	Myc Assay	45	65.0 (58.0–72.7)	57.8 (50.2–65.4)	51.7	50	-	[100]
Springer et al.	Hematological malignancies	213 (2128)	9 *	100%	Serum–single ^§§^	Real time PCR	-	100.0 (39.8–100.0)	27.4 (17.6–39.1)	100	7.0	161	[15]
					Serum–multiple ^§§^			50.0 (6.8–93.2)	52.1 (40.0–63.9)	95.0	5.4	1.1	
Lass-Flörl et al.	Hematological malignancies or SOT	36 (205) ***	24 **	100%	Whole blood	Traditional	34	44 ^§^	100 ^§^	58 ^§^	-	-	[31]
Springer et al.	Hematological malignancies	46	3 *	100%	Whole blood–single ^§§^	Real time PCR	60	55	75	64	67	-	[101]
					Whole blood–multiple ^§§^			27	100	60	100		
Buchheidt et al.	Hematological malignancies	218 (847)	33 **	0% ^3^	Serum	Nested	5 CFU/mL	91.7	81.3	98.0	49.3	-	[102]
Da Silva et al.	Hematological malignancies	172 (1311)	20 *		Whole blood	Traditional	35	75.0 (50.6–90.4)	91.9 (86.5–95.3)	97.0	51.7	-	[103]
Badiee et al.	Hematological malignancies	62 (230)	10 *		Serum	Nested	1 CFU/mL	80	96.2	88.9	92.6	-	[104]
Springer et al.	Hematological malignancies	213 (2128)	17 *		Serum-single ^§§^	Real time PCR	60	92.9 (66.1–99.8)	73.1 (61.8–82.5)	98.3	38.2	34.5	[15]
					Serum-multiple ^§§^			71.4 (41.9–91.6)	92.3 (84.0–97.1)	94.7	62.5	29.4	

* proven/prob according to EORTC 2008. ** proven/prob according to EORTC 2002. *** only pts with proven/prob/poss IPA included. ^§^ for probable IPA. ^§§^ single = one positive test required; multiple = two or more positive tests required. ^#^ at time of PCR. ^1^ negative likelihood ratio. ^2^ positive likelihood ratio. ^3^ explicitly mentioned that NO antifungal prophylaxis was administered.
